# Neuromodulation and Habituation: A Literature Review and Conceptional Analysis of Sustaining Therapeutic Efficacy and Mitigating Habituation

**DOI:** 10.3390/biomedicines12050930

**Published:** 2024-04-23

**Authors:** Anand S. Patil, Brittni Levasseur, Mayank Gupta

**Affiliations:** 1 St. Luke’s Rehabilitation Medical Center, Spokane, WA 99202, USA; 2 Neuroscience Research Center, LLC, Overland Park, KS 66215, USA; 3 Kansas Pain Management, Overland Park, KS 66210, USA

**Keywords:** spinal cord stimulator, neuromodulation, explant, chronic pain, waveform

## Abstract

Spinal cord stimulation (SCS) is a therapeutic modality for the treatment of various chronic pain conditions that has rapidly evolved over the past 50 years. Unfortunately, over time, patients implanted with SCS undergo a habituation phenomenon leading to decreased pain relief. Consequently, the discovery of new stimulation waveforms and SCS applications has been shown to prolong efficacy and reduce explantation rates. This article explores various SCS waveforms, their applications, and proposes a graded approach to habituation mitigation. We suspect the neural habituation phenomenon parallels that seen in pharmacology. Consequently, we urge further exploration of the early introduction of these stimulation strategies to abate spinal cord stimulation habituation.

## 1. Introduction

Chronic pain is a debilitating condition that affects the quality of life of approximately 51 million adults in the United States (20.5% of the population). Its economic impact is estimated to range from USD 560 to USD 635 billion per year in healthcare expenditure and lost economic value production [1,2,3]. The prevalence of chronic pain—specifically chronic back pain—continues to increase, outpacing the prevalence of cancer, heart disease, and diabetes combined [4]. There are a multitude of treatment options for chronic pain, each with their own associated risks, thus making chronic pain management a complex undertaking. Current options include pharmacological therapy, surgery, minimally invasive procedures, physiotherapy, psychological and behavioral treatments, or multimodal treatment [5]. Specifically, within the past few decades, there has been a rise in the use of spinal cord stimulators (SCS) as a treatment option for chronic pain patients—approximately 34,000 SCS are implanted annually around the world [6].

The first spinal cord stimulator was implanted to alleviate cancer-related pain in 1967 by Shealy and colleagues [7]. In the following years, SCS technology has evolved considerably and is considered a minimally invasive treatment for a wide array of indications. Most commonly, implantations are performed for chronic, intractable pain secondary to failed back surgery syndrome (FBSS), complex regional pain syndrome (CRPS) [8], neuropathy [9], and low back pain not amenable to surgery [10,11]. A spinal cord stimulator is composed of a battery and a pulse generator system that attaches to spinal epidural electrode arrays known as leads. These leads are snaked up the epidural space to the vertebral level, where the nociceptive region receives its neuronal innervation. There, the leads deliver electrical impulses that inhibit the transmission of the pain signals traveling along the spinal cord to the brain [12].

There have been numerous advancements in SCS over the past fifty years, such as improvements in device hardware, electrode technology, and stimulation delivery [13]. In addition, novel stimulation waveform paradigms—differing in frequency, duration (pulse width), and amplitude—have improved efficacy [14]. Although there has been tremendous growth in this field, there is a dearth of studies analyzing the long-term efficacy of SCS. Specifically, patients have reported diminished analgesic effects over time, which is also notably the most common reason for device explantation [15,16,17]. This phenomenon is known as habituation (or tolerance), defined as the development of inadequate pain despite a good initial response and having ruled out hardware-related issues or changes in stimulation coverage [18]. A combination of techniques centered around stimulation waveform modification (deemed salvage therapy) have been proposed to address habituation [19]. In this review, we explore contributors to the habituation phenomenon and posit that the early initiation of waveform variability can decrease the rate of explantation due to therapeutic failure.

## 2. Understanding Spinal Cord Stimulation and Waveforms

The initial indication for spinal cord stimulation discovery in the 1960s was for the treatment of chronic neuropathic pain. Electrical current was delivered as a constant (tonic) to the spinal cord. The efficacy of tonic stimulation is based on the Gate Control Theory of Pain Transmission proposed by Melzack and Wall. This theory states that stimuli to the brain via neural pathways are modulated via “gates” [20]. Generally, pain signals are conducted on primary small-fiber afferent nerves, while other stimuli (e.g., non-pain sensations) are conducted via large-fiber interneurons. If one were to continually stimulate the large-fiber interneurons, there would be a “blockage of the gate”, thus preventing small-fiber afferent transmission of pain signals to the brain.

Important parameters to consider in the delivery of energy by SCS are amplitude, frequency, and duty cycle. Amplitude indicates current intensity; frequency is the rate at which stimulation is delivered; and duty cycle is the ratio of the pulse width to the pulse duration. A variation of these variables has enabled the development of the different waveforms discussed below.

### 2.1. Conventional/Tonic Stimulation

Tonic SCS, also known as conventional SCS (cSCS), capitalizes on this principle by delivering a constant stream of pulses that are perceived as paresthesia or tingling carried by large-fiber interneurons. These sensations effectively block pain perception at a targeted vertebral level. Tonic stimulation is characterized by having a high amplitude above the sensory threshold (3.6–8.5 mA), a low frequency (40–100 Hz), and pulse widths ranging from 300–600 µs [21]. The goal is to obtain a fifty-percent reduction in pain or greater with cSCS—a target achieved by nearly half of all patients implanted [22].

One of the challenges of cSCS is the need for accurate coverage of the nociceptive area while accounting for spinal anatomy and neurophysiology [23,24]. Occasionally, patients report uncomfortable paresthesia or unpleasant stimulation associated with changes in posture [25,26]. With regard to the duration of pain relief, several studies have reported that cSCS provides analgesia in patients with low back and leg pain for six months [15,22] to one year [27]. Similarly, a systematic review published in 1995 reported that 62% of patients had pain relief for upwards of one year after implantation. However, a downtrend in pain relief was observed, with ratings of 53% relief at five years and 35% relief 10 years after the implant [28]. Similarly, a 2002 prospective study reported significant pain relief as far along as two years after SCS implantation. However, at both 30-month and 48-month follow-ups, pain scores were significantly worse [29]. Although the etiology of decreased efficacy has not been identified, repeated, sustained tonic stimulation could possibly contribute to decreased pain-relieving effects over time. Consequently, innovations in stimulation delivery have attempted to address habituation and unwanted effects such as paresthesia.

### 2.2. High-Frequency Stimulation

High-frequency SCS (HF-SCS) is one alternative method used to produce paresthesia-free pain relief. HF-SCS utilizes short-duration pulses of stimulation (30 µs) at high frequency (1–10 kHz) and amplitudes (1–5 mA) below the sensory threshold [30]. Since high-frequency stimulation is below the sensory threshold, patients do not report feeling paresthesia at target sites. This development broadened the scope of SCS application, specifically for the treatment of chronic low back pain, which had previously been elusive to treat with SCS [31]. Several randomized controlled trials have reported superiority of HF-SCS compared to conventional/tonic stimulation in measures such as quality of life, pain intensity, functional outcomes, and decrease in opioid use [17,32]. For example, patients treated with HF-SCS had a 67% decrease in their mean back pain score (as characterized on the Visual Analog Scale for pain intensity) compared to only a 44% decrease in patients treated with cSCS over a twelve-month period [17]. Furthermore, HF-SCS has been reported to have clinically significant and sustained pain relief one [33], two [32,34], and three years [35] after implantation.

### 2.3. Burst Stimulation

Burst stimulation programming was introduced by De Ridder and colleagues in 2010. It cycles through a short interval of high-frequency (500 Hz) stimulation, followed by pulse-free charge phases, and then a rest phase for recharge [36]. The rest phase is thought to mimic the natural neuronal firing pattern involved in pain processing, thus allowing for exogenous control of pain signals free of paresthesia. The clinical efficacy of burst SCS on various pain conditions has been reported in several prospective and cohort-designed studies, as noted in Kirketeig et al. [37]. Similar to HF-SCS, burst SCS has been reported to be superior in pain relief and preferred by patients compared to cSCS [38,39]. In 2016, De Ridder et al. reported that burst stimulation was able to improve back, limb, and general pain by 51%, 53%, and 55%, respectively, compared to 30%, 52%, and 31%, respectively, by tonic stimulation [40]. Small sample observational studies have compared burst stimulation and HF-SCS in reducing pain scores in individuals suffering from low back pain. Two studies reported equivalent immediate efficacy in back and leg pain reduction between high frequency and burst waveforms [41,42]. Of note, burst SCS was superior to HF-SCS in reducing leg pain at 3 months [41] and 12 months [42]. Interestingly, burst stimulation was also noted to have positive effects on patient affect and emotions. Researchers posit that this may be due to neuromodulation of brain regions involved in pain processing [40,43,44].

### 2.4. Intermittent Dosing Burst Paradigm

The superiority of burst SCS as compared to cSCS has been well established. In an effort to make burst stimulation more efficient, researchers set forth to investigate if limiting the amount of time burst stimulation is active (ON) and inactive (OFF) (duty cycle) can provide a sustained therapeutic effect. The benefit would be twofold: possibly increased time before habituation and decreased energy consumption for stimulation [45]. In 2019, Vesper et al. compared burst SCS to intermittent dosing (also known as microdosing) burst SCS in alternating cycles of five seconds ON and five to ten seconds OFF. They found no significant difference in reported pain relief or quality of life [45]. This was further investigated by Deer et al., who had patients undergo a trial pattern of thirty seconds ON and ninety seconds OFF. Patients were then instructed to select the longest tolerable OFF period that achieved pain control similar to that of the trial period (90, 120, 150, 240, or 360 s). After six months, 45.8% of patients were using the 360 s OFF period, and the remainder of the patients were evenly distributed among the shorter OFF periods. Comparing the different prolonged OFF period groups amongst each other and to the trial period, patients did not report significant exacerbations of pain, a decrease in quality of life, or increased pain catastrophizing [46]. Therefore, this study demonstrates that intermittent burst SCS can be customized to provide optimal pain relief and reduce the amount of stimulatory electrical current. Unlike cSCS, the long-term efficacy of intermittent burst SCS has not been thoroughly studied.

## 3. Closed-Loop Spinal Cord Stimulation

As previously discussed, the electrodes at the ends of the leads are fixed in the epidural space. However, the spinal cord is constantly changing position with even the smallest of physical movements (e.g., breathing, coughing, sitting, and standing) [47]. These movements cause changes in the distance between the electrode and spinal cord, resulting in large changes in the amount of current transmitted to neural tissue [48,49]. This is known as an open-loop system; there is no autofeedback mechanism for the stimulator to adapt to positional changes. The risk is the delivery of inconsistent therapy with the potential for unwanted side effects or, more significantly, loss of efficacy leading to explantation [50]. To address these limitations, a closed-loop neuromodulation system was developed to continuously measure electrophysiological and postural changes and adjust the stimulation dose [51].

The safety and efficacy of the closed-loop SCS system in patients with leg and low back pain have been reported in several studies [50,51]. Russo et al. reported that at twelve months post-implantation, more than seventy-five percent of patients had ≥50% back, leg, and overall pain relief, and more than half of these patients had ≥80% pain relief [52]. Additionally, the superiority of closed-loop to open-loop SCS as measured by improvement in leg and back pain relief has been demonstrated. Mekhail et al. reported that a greater proportion of closed-loop SCS patients had ≥50% relief in overall pain compared to open-loop patients at three months (82.3% vs. 60.3%, respectively), twelve months (83.1% vs. 61.0%, respectively), and twenty-four months (79.1% vs. 53.7%, respectively) post-implantation [48,50]. Additionally, closed-loop SCS has demonstrated marked improvements in patients’ health-related quality of life, physical and emotional functioning, and a reduction or complete elimination of opioid use at 24 months [51,53].

## 4. Habituation and Spinal Cord Stimulator Explantation

Although SCS is an effective therapeutic option for various chronic pain conditions, rates of explantation have been noted to increase after the first year of implantation [54,55]. In an observational, nonrandomized, retrospective study of over seventy-nine thousand Medicare beneficiaries who received rechargeable SCS from 2013 to 2020, at one, three, five, and seven years post-implantation there was a 5.1%, 12.5%, 17.6%, and 22.0% cumulative incidence of explantation, respectively [55]. One of the most frequent reasons for explantation is inadequate pain control. Simopoulos et al. reported that in a sample pool of two hundred fifty-two SCS patients, a total of 30% had their device removed, with 28% of those undergoing removal citing ineffective pain control as the reason [56]. Further, Van Buten et al. reported that there was a 7.9% annual explantation rate in a study of nine hundred fifty-five SCS patients. Half of those patients reported inadequate pain relief prior to explantation [57]. More recently, a study by Al-Kaisy et al. performed a retrospective analysis on 182 patients implanted with a neuromodulation device such as SCS and reported an explantation rate of 17.8% at 5 years and 25.2% at 10 years. The most common reason for explantation was loss of efficacy (65%), with rates of explantation reported to be 13.3% at 5 years and 17.5% at 10 years, regardless of indication [58].

Individuals that report loss of efficacy from SCS are thought to experience habituation, which is described as the progressive loss of pain control despite initial analgesic success and appropriate stimulation that cannot be explained by hardware-related issues [16,59]. Habituation has been reported to affect 13–25.9% of SCS patients [60]. The biological etiology of habituation is not well understood. Many physiological, pathological, and psychological components have been suggested, with neuronal plasticity being a major contributing factor [19,61,62]. The onset of therapy habituation is influenced by time, type of SCS device, and the patient’s pain condition [63]. For example, Levy et al. reported that tonic SCS demonstrated therapy habituation as early as nine to twelve months in patients with CRPS [62].

Pharmacological sensitization is a unique process; understanding its etiology may shed light on further understanding habituation caused by persistent stimulation of the nerve. Tolerance is defined as the reduction in or loss of drug efficacy in the setting of long-term use [64]. Per the United States Food and Drug Administration guidelines, a patient is considered tolerant to opioids at doses greater than 60 mg morphine equivalents (MME) per day [65]. As summarized in Li et al., mechanisms contributing to opioid tolerance include increased drug metabolism, the compensatory/opposing process, and the downregulation of opioid receptors [66].

Similarly, it is speculated that prolonged exposure of neuronal tissue to stimulation patterns causes a tolerance phenomenon to occur secondary to repetitive driving of electrophysiological and neurochemical mechanisms. If a patient experiences drug tolerance, their physician may increase the dose or recommend a new medication [67]. Therefore, in an attempt to regain pain relief after the habituation of SCS and loss of efficacy, several studies have examined replacing the original stimulation waveform (which has been adapted to) with a new SCS waveform or program.

Interestingly, Rauck et al. reported that 7.6% of a cohort of one thousand two hundred eighty-nine SCS subjects using custom and variable stim programming required an explant three years post-implantation, with only 2.5% of these subjects undergoing explanation due to inadequate pain relief [68]. This markedly lower incidence of overall explantation due to habituation compared to other studies could be partially explained by the induction of different cellular mechanisms of analgesia controlled by variable-stimulation programming [68]. Further, several studies have demonstrated that patients who had not maintained pain relief with conventional SCS therapy achieved pain improvement when their traditional SCS was replaced with HF-SCS [69,70,71]. Kapural et al. reported that 81% of patients who were switched to HF-SCS due to cSCS habituation received greater than 50% pain relief with 10 kHz stimulation [69]. Similarly, Cordero Tous et al. demonstrated improvement in analgesia after transitioning from conventional to HF stimulation without replacing existing spinal electrodes. This decreased the complications associated with a second procedure [72]. Provenzano et al. studied thirty-one patients on high-frequency stimulation with varying duty cycles ranging from 3%, 14%, 50%, and 100%. Findings showed that some patients achieved equivalent pain relief with lower duty cycles, which reduced device charging time by approximately two-thirds [18]. Improved pain relief has also been reported in patients who were previously implanted with cSCS and then received burst stimulation. Courtney et al. reported that 76% of patients who had been using tonic stimulation for at least 90 days had a reduction in overall daily pain intensity after using burst stimulation for two weeks [73].

Another rescue strategy that has proven to be effective is incorporating a “stimulation holiday,” during which SCS therapy is discontinued for a period of time prior to restarting [61]. D’Souza et al. reported that 57.5% of patients that underwent a stimulation holiday (17.3 ± 20.6 days) after experiencing a loss of efficacy with HF-SCS had ≥50% relief in pain intensity with maintenance of pain relief for 6 months [61]. Akin to receptor degradation with overexposure to inhibiting medications in pharmacology, allowing for a stim holiday allows the intrinsic responsivity and receptor-mediated pathways to reset, thus re-establishing sensitivity [74,75,76].

## 5. Conclusions and Future Directions

Chronic pain affects millions of adults in the United States and disrupts nearly all aspects of an individual’s life. The field of SCS has evolved tremendously since its first use in 1967, particularly with the addition of several different stimulation waveforms. Given the novelty of the therapy, long-term data on these various SCS waveforms is sparse. Several studies have reported a high incidence of SCS explants, with loss of efficacy being one of the primary reasons for removal. Although several strategies, such as stimulation holidays [61], alternating waveforms [69,72], or SCS microdosing [45], have been proposed to regain pain relief, there has yet to be an established methodology to abate habituation and significant loss of efficacy. In 2023, Mirzakhalili et al. published “An optimization framework for targeted spinal cord stimulation.” They developed computer modeling, which allowed for customization of stimulation configurations for targeted SCS. Furthermore, the computational model can be integrated into individual patient variables and allow for more efficient and personalized therapy [77].

The MULTIWAVE study, a prospective, randomized, controlled, crossover, double-blind trial, showed that allowing switching of waveforms and/or combining waveforms increased the rate of SCS responders by 25%. This study also found waveform versatility had a positive and sustained response in patients who had been salvaged from cSCS loss of efficacy [78]. Given the current evidence implicating the individual effectiveness of SCS microdosing [45], turning stimulation off for a designated period [61], pulse dosing, and cycling between different stimulation waveforms [45,46], we hypothesize that implementing all three strategies immediately after SCS implantation will prolong device efficacy and pain relief. A summary of these technologies and their benefits can be appreciated in Table 1. These stimulation strategies can be viewed as steps of habituation mitigation that work in tandem to ultimately extend significant pain relief duration post-SCS implantation.

**Figure 1:**
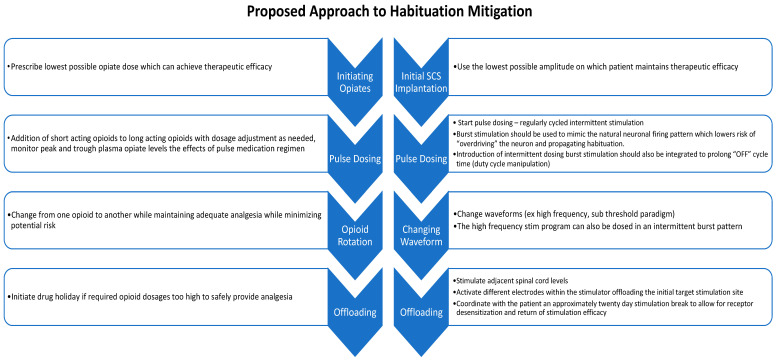
Graded approach to pharmacological and stimulation habituation mitigation.

In Figure 1, we propose a graded approach to limiting habituation, closely paralleling the interventions that are known to work with opiate tolerance. We acknowledge that the exact biological mechanism of spinal cord stimulation has yet to be clearly elicited; however, we aim to establish a framework to approach habituation phenomena. Further prospective trials are encouraged to validate this proposed approach. Changes in stimulation programming are compared to equivalent approaches in opioid therapy, as we suspect electrostimulation habituation mirrors the habituation phenomenon appreciated in pharmacology. Specifically, more exploration is needed in the deployment timeline of each paradigm and its utility for responder longevity or use as salvage therapy. Therefore, we encourage providers to investigate further strategies for incorporating diverse stimulation paradigms to abate stimulation habituation.

## Figures and Tables

**Table 1:** Summary of stimulation paradigms.

Stimulation Paradigm	Mechanism (Altered Parameter)	Benefit
**High Frequency Stimulation**	High frequency of stimulation ranging from 1–10 kHz with amplitudes below the sensory threshold	No parasthesia at target sites [30], greater decrease in mean back pain score compared to cSCS [17], sustained pain relief up to three years after implant [35]
**Burst Stimulation**	High frequency stimulation period followed by rest phase thought to mimic natural neuronal firing pattern [36]	Notable improvement in back, limb and general pain compared to cSCS [40], superior to HF–SCS in reducing leg pain [41,42], positive effects on mood [40,43,44]
**Intermittent Dosing Burst Stimulation**	Duty cycle alteration (amount of time burst stimulation is active and inactive)	No difference in pain relief or quality of life compared to burst [45], increasing time to habituation due to overall decrease in time nerve is stimulated [45], decreased energy consumption of system [45], increased customizability to patient preference [46]
**Closed Loop System**	Constant measure and response to changing electrophysiological and postural changes (ie more efficacious stimulation to target) [51]	Greater pain relief as compared to open-loop systems from three months to two years [48,50], improvement in patient quality of life, emotional functioning and reduciton in opiate use at two years [51,53]
**Stimulation Holidays**	Discontinue stimulation for a period of time before restarting, thought to reset receptor mediate pathways and re-establish sensitivity [74,75,76]	Approximatley 20 day holiday can lead to significant, sustained pain relief 6 months later [61]
